# Willing to wait?: The influence of patient wait time on satisfaction with primary care

**DOI:** 10.1186/1472-6963-7-31

**Published:** 2007-02-28

**Authors:** Roger T Anderson, Fabian T Camacho, Rajesh Balkrishnan

**Affiliations:** 1 Department of Public Health Sciences, Wake Forest University Health Sciences, Medical Center Boulevard, Winston-Salem, NC, 27157 USA; 2 College of Pharmacy and School of Public Health, The Ohio State University, Columbus, OH, 43210 USA

## Abstract

**Background:**

This study examined the relationship between patient waiting time and willingness to return for care and patient satisfaction ratings with primary care physicians.

**Methods:**

Cross-sectional survey data on a convenience sample of 5,030 patients who rated their physicians on a web-based survey developed to collect detailed information on patient experiences with health care. The survey included self-reported information on wait times, time spent with doctor, and patient satisfaction.

**Results:**

Longer waiting times were associated with lower patient satisfaction (p < 0.05), however, time spent with the physician was the strongest predictor of patient satisfaction. The decrement in satisfaction associated with long waiting times is substantially reduced with increased time spent with the physician (5 minutes or more). Importantly, the combination of long waiting time to see the doctor and having a short doctor visit is associated with very low overall patient satisfaction.

**Conclusion:**

The time spent with the physician is a stronger predictor of patient satisfaction than is the time spent in the waiting room. These results suggest that shortening patient waiting times at the expense of time spent with the patient to improve patient satisfaction scores would be counter-productive.

## Background

The literature on patient satisfaction with primary care indicates that key attributes of health care valued by patients are patient-centered, including time spent with the physician, willingness of the physician to listen to the patient, and expectations for treatment [1-4]. An anecdotal source of dissatisfaction with health care reported by patients is having to wait a long period of time in the office (Anderson, Barbara, Feldman, in press [5]), and several studies have documented the negative association between increased waiting time and patient satisfaction with primary care [6-10]. However, waiting time is but one aspect of health care that patients' value, and its centrality to patients' assessment of their primary care visit compared to other aspects of the health care experience is uncertain. Time spent waiting is a resource investment by the patient for the desired goal of being seen by the physician and therefore may be moderated by the outcome. In a typical practice, patient waiting time and time spent with the physician are to some degree counter-controlled. Since the amount of daily clinic time per physician is a fixed asset, portioned out by patient demand or volume, the more time on average a specific physician spends with an individual patient, the longer will patients have to wait to see that physician. This leads to the testable hypothesis that the effect of waiting time on patient satisfaction must be considered in the context of time spent with the patient to be meaningful. If our hypothesis is correct, physicians who fall behind in their patient schedules and end up having both long patient wait times and shorter visits with the patient will achieve significantly lower patient satisfaction scores than physicians who have both long patient wait time and long patient visit times.

## Methods

This study was conducted from the responses of a national cross-sectional, online survey of patient's satisfaction (DrScore.com) that collected anonymous patient ratings of U.S. primary care physicians for patient advocacy research and to produce patient satisfaction report cards for physicians. The survey focused on the most recent outpatient visit and used a list of U.S. physicians that permitted patients to look up their doctors and access the survey. Participation in the survey was advertised to patients on a public radio show (The Peoples Pharmacy), through patient advocacy groups, and through on line search engines. The survey asked patients to both rate their physician on several dimensions of health care experiences, as well as provide specific comments about aspects of care that were most excellent or most in need of improvement. Questions were rated on a scale of 0 ('not al all satisfied') to 10 ('extremely satisfied'). Two patient satisfaction scores were considered as outcomes in this study: ratings of the provider (Physician Care, 9 items) on the thoroughness of care, physician communication and follow-up, listening, demeanor, discussion of test results, answering questions, treatment success, and including the patient in decision processes; a second rating was of the practice (Office Practice, 5 items) and included items on continuity of care, convenience of facility, referrals, hours, and ability to meet health care needs of the patient. For both scales, the summed scores were scaled from 0 to 100 by taking the item mean and multiplying it by 100, representing compete satisfaction on all characteristics measured.

Patient waiting time at the last office visit was measured by asking the patient to recall the amount of time he/she waited before being seen by the physician for a scheduled appointment. Response categories were: 1–5 minutes waiting time in office, 6–15 minutes, 16–30 minutes, 31–60 minutes, and more than 1 hour. The shorter time intervals at the start were chosen because pilot data showed that approximately 70% of the patients waited below 15 minutes. Perceived time spent with the physician was measured as < 5 minutes, 6–10 minutes, and > 10 minutes, also assessed by patient recall. No personal identifying information were collected in this study (e.g., name, address, of medical number) and expedited IRB approval was obtained to conduct analyses of de-identified data.

### Statistical analysis

Multivariate regression and logistic regression models predicting the three satisfaction ratings were estimated using the Generalized Estimating Equations (GEE) method implemented in the SAS System v9 (GenMod procedure)[11] In order to adjust for clustering, an exchangeable working correlation matrix was specified where the observations were clustered according to clinic. The default robust standard errors in proc GenMod were used. All models were adjusted for patient reported age, gender, reason for visit, and first visit. Age was modeled as a continuous variable based on its observed close approximation to a linear response to an overall rating of patient satisfaction with physician seen. Assessments of covariates such as type of health care organization, severity of illness, and race were not collected in the study survey.

## Results

Table 1 displays the descriptive characteristics of the study sample. On a scale of 0–100 (highest), the mean satisfaction with doctor score with the overall practice were each approximately 74. The majority (roughly 60%) were ages 25 to 44, 74 percent were female. Of the visits rated, 13.5% were a first visit, 28.4% were for routine evaluation or management. Approximately 25% of respondents reported that they waited more than 30 minutes to be seen; and 11% reported spending less than 5 minutes with the doctor, 27% spent between 5 and 10 minutes, while 62% reported spending more than 10 minutes with their doctor.

**Table 1:** Baseline Characteristics of the Study Population

**Parameter ** **↓**	**Case Group (N = 5003) Mean (SD) [Range]**
Doctor Care Score	73.62 (35.89) [0–100]
Practice Care Score	73.96 (30.02) [0–100]
Age group (%)	
Less than 18	6.76%
18 – 24	21.87%
25 – 34	24.27%
35 – 44	36.78%
45 – 64	10.01%
65 +	0.32%
Male Gender (%)	25.70%
First visit to office (%)	13.47%
Routine exam or check-up	28.40%
Wait Time category	
Less than 15 min	37.92%
15 to 30 min	37.56%
30 to 60 min	14.89%
60 min +	9.63%
	25.22 (20.47)
Visit Time category	
Less than 5 min	11.11%
5 to 10 min	26.86%
10 min +	62.02%

In univariate analyses, time spent with the physician was found to be (Spearman rank) correlated with overall patient satisfaction rating at r = .51 compared to r = .31 for waiting time in the office. In Table 2 are the results from the multivariable regression analysis for the Doctor rating scale considering all model predictors. Overall, 43% of the variance in patient satisfaction was explained by the final set of predictors (p < .05) included: age, first visit, reason for being seen (routine versus other reasons), waiting time, and visit time. A similar set of predictors and results was obtained for the Practice scale score. Each of the latter variables were independently associated with patient satisfaction (adjusting for all other factors). Those with a first visit with physician were each associated with lower patient satisfaction than shorter waiting times and longer visit times. Of all variables considered, time spent with the physician was the most powerful predictor of patient satisfaction, explaining 28% of the variance, almost 3 times larger than waiting times (data not shown).

**Table 2:** Predictors of Doctor Rating using Mixed Model Regression (N = 5003)^§^

***Dependent Variable***⇒ **Predictor Variables **⇓	**Doctor Care Score β (se)**
Intercept	37.00 (1.74)***
Age^§§^	0.082 (0.036)*
Male Gender	-0.22 (0.88)
First visit to office	-12.57 (1.74)***
Routine exam or check-up	4.97 (0.84)***
Waiting time^§§^	-0.39 (0.021)***
Visit time^§§^	3.78 (0.089)***
Interaction First visit with Waiting time	-0.14 (0.049)**
Adjusted R^2^	0.43

Notes:

*P < 0.05 level, **P < 0.01, *** *P *< 0.001

^§ ^Includes a random effect for subject.

^§§ ^Continuous approximation.

**Figure 1:**
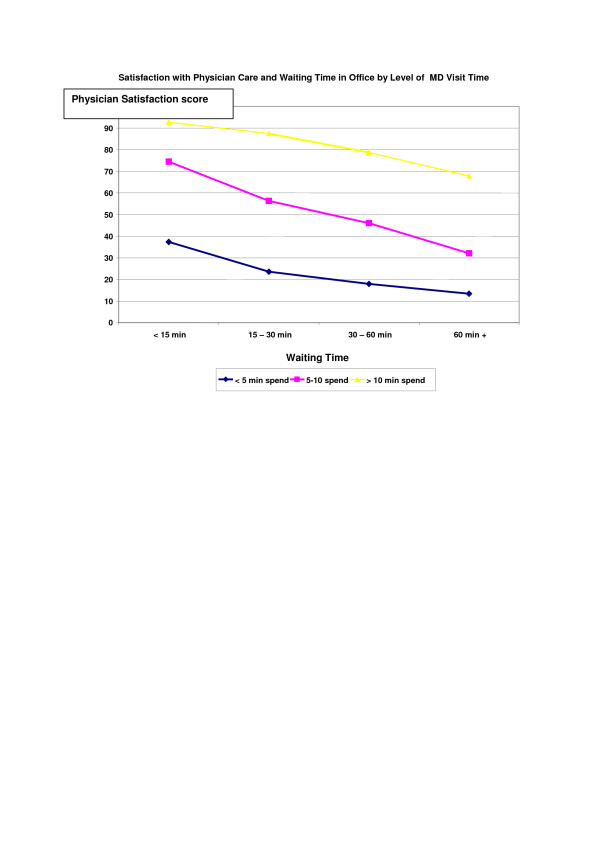


**Figure 2:**
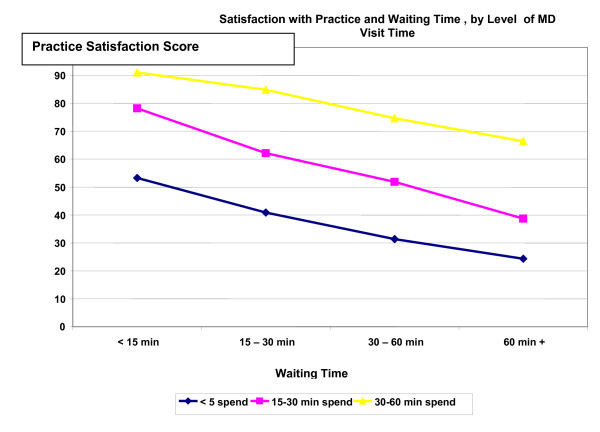


The relationship of waiting time, the primary focus of this study, on the patient satisfaction ratings was found to be moderated by time spent with the physician. Figure 1 displays perceived waiting time effects by levels of perceived time spent with physician. For example, among those who reported waiting 30–60 minutes to see their physician, the mean Physician care scale score was 18.0 when < 5 minutes was reported spent seeing the physician versus 78.7 when > 10 minutes was spent with the physician. The most satisfied patients were those who had brief waits (< 15 minutes) and longer visits, with a mean Physician Care satisfaction score of 92.7. The same general pattern of results is true as well for the Practice mean (Figure 2).

## Discussion

This study is among the first to examine the relationship of patient reported waiting times and visit time on overall patient satisfaction. We found that of the two time-based measures, time spent with the physician is most powerful determinant of overall patient satisfaction. However, the combination of long wait times and short visit times produced the lowest level of patient satisfaction observed in the study, and suggests that both measures are important. This suggests that clinics facing operational constraints on physician staffing concurrently with high patient loads, will face accelerating patient dissatisfaction as physicians reduce time spent with patients and patients have to wait longer to be seen. Patients currently give physicians considerable leeway in waiting times as long as they feel they get adequate time with their physician. Our study suggests that long waiting times and short visit times are a toxic combination for patient satisfaction and one that providers and practice mangers should avoid if they are concerned about patient-centered measures of health care quality such as patient satisfaction. While having sufficient visit time with the physician is of paramount importance, we hypothesize that short visits with the physician are more negatively valued as waiting time increases because the patient's resource investment (time) is higher and is likely appraised as a poor trade for the obtained outcome. There was no evidence of an interaction of wait times and visit times. Thus the results displayed represent additive effects of both time-derived variables rather than buffering, or effect modification. Still, the cumulative effects of waiting time upon patient satisfaction are influenced by  physician visit time. There are several limitations of this study to consider. First, the internet survey likely resulted in a biased sample by selectively attracting respondents who were experienced using the internet and willing to complete a survey regarding patient satisfaction. The survey completion 'response rate,' comprised of those who accessed the survey site and chose to complete the survey is not known, but is likely to be low. To this extent, the data and results may not be generalizable to the larger population of patients in the community. To gauge the robustness of our results across settings, we examined the effects of waiting time and visit time in a companion study we have recently completed on drivers of patient satisfaction in a large academic primary care organization (Camacho et al, in press[12]). The latter study achieved approximately an 80% response rate using a survey delivered at the point of care, among N = 2535 patient volunteers. We found in this newer study that both waiting time and visit time were significant predictors of overall patient satisfaction, and willingness to return for care. Another limitation is that this study assessed self-reported wait and visit times. It is possible that recall basis is present such that satisfaction with the overall doctor visit influenced the perception of time spent. To this extent, the link between time and satisfaction may be spurious. However, a comparison performed by Dansky[6] between actual and perceived waiting times shows only a slight overestimation with no significant differences (using 323 subjects) between both measures. Finally, we did not examine effects or correlation from health care system variables such as type of primary care physician, health plan or organization model

## Conclusion

The time spent with the physician is a stronger predictor of patient satisfaction than is the time spent in the waiting room. These results suggest that shortening patient waiting times at the expense of time spent with the patient to improve patient satisfaction scores would be counter-productive.

## Competing interests

The lead author (RA) serves as a Director of DrScore, a privately held patient satisfaction research firm which supplied data for this study, and has received no financial compensation for this work. The co-authors have no competing interests to declare.

## Authors' contributions

RA led the design and conceptualization of the study, and the results report. FC performed the analyses and contributed to the written manuscript. RB contributed by refining the manuscript and measurement models. All authors read and approved the final manuscript.

## Pre-publication history

The pre-publication history for this paper can be accessed here:


